# An analysis of codon utilization patterns in the chloroplast genomes of three species of *Coffea*

**DOI:** 10.1186/s12863-023-01143-4

**Published:** 2023-08-09

**Authors:** Yaqi Li, Xiang Hu, Mingkun Xiao, Jiaxiong Huang, Yuqiang Lou, Faguang Hu, Xingfei Fu, Yanan Li, Hongyan He, Jinhuan Cheng

**Affiliations:** 1 Institute of Tropical and Subtropical Cash Crops, Yunnan Academy of Agricultural Sciences, Baoshan, Yunnan China; 2 Institute of Tropical Eco-Agricultural, Yunnan Academy of Agricultural Sciences, Yuanmou, Yunnan China; 3 Yunnan Academy of Agricultural Engineering, Kunming, Yunnan China

**Keywords:** Coffee, Chloroplast genome, Codon usage bias, Heterologous expression

## Abstract

**Background:**

The chloroplast genome of plants is known for its small size and low mutation and recombination rates, making it a valuable tool in plant phylogeny, molecular evolution, and population genetics studies. Codon usage bias, an important evolutionary feature, provides insights into species evolution, gene function, and the expression of exogenous genes. Coffee, a key crop in the global tropical agricultural economy, trade, and daily life, warrants investigation into its codon usage bias to guide future research, including the selection of efficient heterologous expression systems for coffee genetic transformation.

**Results:**

Analysis of the codon utilization patterns in the chloroplast genomes of three Coffea species revealed a high degree of similarity among them. All three species exhibited similar base compositions, with high A/T content and low G/C content and a preference for A/T-ending codons. Among the 30 high-frequency codons identified, 96.67% had A/T endings. Fourteen codons were identified as ideal. Multiple mechanisms, including natural selection, were found to influence the codon usage patterns in the three coffee species, as indicated by ENc-GC3s mapping, PR2 analysis, and neutral analysis. *Nicotiana tabacum* and *Saccharomyces cerevisiae* have potential value as the heterologous expression host for three species of coffee genes.

**Conclusion:**

This study highlights the remarkable similarity in codon usage patterns among the three coffee genomes, primarily driven by natural selection. Understanding the gene expression characteristics of coffee and elucidating the laws governing its genetic evolution are facilitated by investigating the codon preferences in these species. The findings can enhance the efficacy of exogenous gene expression and serve as a basis for future studies on coffee evolution.

**Supplementary Information:**

The online version contains supplementary material available at 10.1186/s12863-023-01143-4.

## Background

Coffee, a tropical and subtropical evergreen shrub or small tree, is cultivated in tropical regions of Asia and Africa. It is one of the three major beverage-producing plants in the world [1]. Coffee has become the second most valuable commodity globally, following oil, [2]. Commercially grown coffee consists of three main species: *Coffea arabica*, *Coffea canephora*, and *Coffea liberica* [3]. Coffee is rich in protein, crude fiber, crude fat, and caffeine, among other compounds. It has gained popularity as an everyday beverage due to its distinct aroma and invigorating effects [2]. Additionally, coffee holds significant therapeutic value, including liver protection, bowel regulation, constipation relief [4, 5], immune-boosting properties, prevention of chronic illnesses [6, 7], regulation of fat metabolism, reduction of blood lipids, and cancer prevention [2, 8].

The codon, as the primary carrier of precise genetic information, plays a crucial role in biological heredity and variation in nature. Except for methionine and tryptophan, the remaining 20 amino acids in natural proteins correspond to 2–6 codons, known as synonymous codons [9]. It has been observed that different organisms exhibit preferential selection of specific synonymous codons for encoding amino acids [10]. Moreover, codon usage frequency can vary among different genes within the same species, this phenomenon was called as codon usage bias [10]. With the advancement of high-throughput sequencing technology and the availability of genomic information for various organisms, investigations and comparisons of codon bias have been conducted for numerous species, including forty *Theaceae* sp [11], five *Miscanthus* sp and related sp [12], and six *Euphorbiaceae* sp [13]. Codon bias can influence protein translation speed and accuracy, mRNA transcriptional regulation, and the expression of exogenous genes [14, 15]. Significant differences in codon usage between exogenous and host genes can impact the translation and expression of the exogenous gene [16]. Therefore, studying codon bias is of great importance for understanding gene function, protein structure, and enhancing the expression efficiency of exogenous genes [16, 17].

The chloroplast, responsible for photosynthesis and biosynthesis of various metabolites such as amino acids, starch, fatty acids, and pigments, plays a crucial role in plant physiology [18]. Chloroplasts possess several advantageous features, including a small genome, stable structure, high conservation, and maternal inheritance. These characteristics have led to their extensive use in plant diversity studies, phylogeny, DNA barcoding, investigations of plant adaptability, and genetic engineering research [19, 20]. Maternal inheritance of chloroplast DNA provides advantages over nuclear transformation systems, such as efficient expression of exogenous genes, high security, multi-gene co-expression, positional independence, and gene silencing [21–24]. Chloroplast engineering has been applied in various fields, including plant resistance [25, 26], high expression of medicinal proteins [27, 28], and agronomic trait improvement [29].

Although the chloroplast genomes of coffee have been sequenced, a comprehensive analysis of codon usage patterns encoded by the chloroplast genome is lacking, providing an important research direction. Therefore, this study systematically analyzes the codon usage patterns and factors influencing them in the chloroplast genomes of three coffee species using the available whole-genome information. It also determines the optimal codons. Furthermore, the comparison of coffee chloroplast genome codons with those of Arabidopsis thaliana, Nicotiana tabacum, Saccharomyces cerevisiae, and Escherichia coli provides a foundation for gene function verification, evolutionary genetics, and chloroplast genetic engineering in coffee.

## Results

### Characteristics of codon utilization bias analysis of codon base composition

The codon usage bias in the chloroplast genomes of three coffee species was investigated by analyzing the GC1, GC2, GC3, and the average GC content (Table 1). It was observed that in all three coffee species, the frequency of GC1, GC2, and GC3 and the average GC content were below 50%, indicating a preference for A/T-ending codons. Additionally, GC1 had a higher frequency than GC2 and GC3, with GC3 showing the lowest frequency. The average GC content of the three coffee species was similar, with values of 38.39%, 38.25%, and 38.11% for the respective species. Notably, *C. liberica* and *C. canephora* exhibited the most similar average GC content, suggesting a high degree of codon preference for both species.

**Table 1:** The values of GC1, GC2 and GC3 of the three coffee species. GC1, GC2 and GC3 represent the GC content at the first, second and third position

Gene group	*Coffea Arabica*	*Coffea canephora*	*Coffea liberica*
GC1%	47.30	47.48	47.37
GC2%	39.07	39.55	39.35
GC3%	27.97	28.14	28.03
The average of GC%	38.11	38.39	38.25

### RSCU and RFSC

The analysis of codon usage bias was performed using RSCU and RFSC measures. There were 30 codons with RSCU > 1 in each of the three coffee genomes, and there were 30 common preference codons of the three coffee genomes. Among these codons, 29 had A/T endings, accounting for 96.67% of the entire quantity of preference codons, it is distinctly more than the amount of preference codons with G/C endings (Table S1). This indicates a clear preference for A/T-ending codons in the three coffee genomes. There were 18 high-frequency codons in the three coffee genomes, which are GCT, TGT, GAT, GAA, TTT, GGA, CAT, ATT, AAA, TTA, AAT, CCT, CAA, AGA, TCT, ACT, GTA, and TAT (Table S1). The range of RSCU values obtained for the three coffee genomes was 0.34–1.90, 0.34–1.90, and 0.35–1.91, respectively. Among these values, the codon TTA encoding Leu had a strong preference in all three coffee species (RSCU > 1.8), while the codon AGC encoding Ser had the lowest code utilization preference among the three coffees (RSCU < 0.4) (Table S1). Overall, the types and quantities of preference codons were highly similar among the three coffee genomes, suggesting a consistent pattern of codon usage.

### Determination of optimal codons

Optimal codons were identified based on the criteria of RSCU > 1 and ΔRSCU > 0.08. Table 2 and Table S2 present the results, revealing a total of 56 optimal codons: 20 for *C*. *arabica*, 17 for *C*. *canephora*, and 19 *C*. *liberica*. Among these optimal codons, 23 ended in A, 30 ended in T, and 3 ended in G. There are 14 optimal codons were shared among all three *Coffea* species.

**Table 2:** Optimal codons of the chloroplast genome of three *Coffea* species

Species	Optimal codon number	Optimal codon
*Coffea arabica*	20	AAA, AAT, ACA, ACT, AGA, ATT, CAA, CGT, GAA, GCA, GCT, GGA, GGT, GTT, TAT, TCA, TGT, TTA, TTG, TTT
*Coffea canephora*	17	AAA, ACA, ACT, ATT, CAA, CGT, CTT, GAA, GCA, GCT, GGT, GTT, TAA, TAT, TGT, TTG, TTT
*Coffea liberica*	19	AAA, ACA, ACT, ATT, CAA, CGT, GAA, GCT, GGA, GGT, GTT, TAA, TAT, TCA, TCT, TGT, TTA, TTG, TTT

### Codon utilization frequency

To further explore the codon usage preferences, a comparative analysis was conducted between the three coffee chloroplast genomes and four commonly used exogenous expression hosts (*A. thaliana*, *N. tabacum*, *E. coli*, and *S. cerevisiae*), separately (Table S3). The analysis revealed that compared with *A. thaliana*, *N. tabacum*, *E. coli* and *S. cerevisiae*, there were 15–16 (23.4%-25.0%), 9–10 (14.1%-15.6%), 26–27 (40.6%-42.2%) and 10 (15.6%) codon utilization modes of the three kinds of coffee separately (Table S3). The codon frequency of the three species of coffee had slightly difference from that of *N. tabacum* and *S. cerevisiae*. Nevertheless, the codon frequency of the three kinds of coffee had quite difference from that of *A. thaliana* and *E*. coli. To summarize, the codon usage frequency is closely related to the exogenous expression efficiency of chloroplast genes in coffee plants, the *N. tabacum* and *S. cerevisiae* can be used as the hosts for heterologous expression receptors of three coffee genes.

### Source analysis of variation in codon utilization

#### ENc-plot

**Fig. 1:**
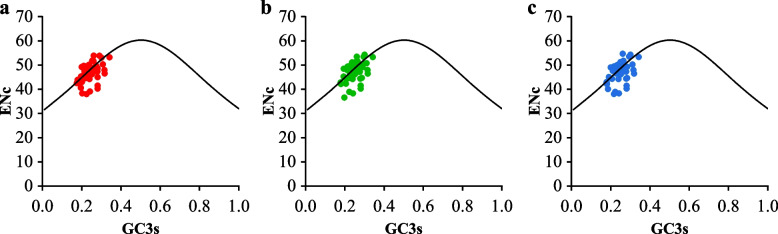
ENc-plot of chloroplast genomes of three Coffea species. **a** *Coffea arabica* **b** *Coffea canephora* **c** *Coffea liberica*

The ENc and GC3s plots were utilized to investigate the major factors influencing codon usage preferences. Figure 1 shows that the ENc-GC3 patterns for the three coffee genomes were similar. Approximately 21–22 (43.75%-44.00%) genes were located on the expected curve, indicating that they closely aligned with the theoretical ENc values. Additionally, approximately 27–28 (56.00%-56.25%) genes fell below the expected curve, deviating significantly from the predicted ENc values. This suggests that while mutation pressure plays a role in codon usage patterns, other factors, particularly natural selection, have a significant impact.

#### PR2-plot

**Fig. 2:**
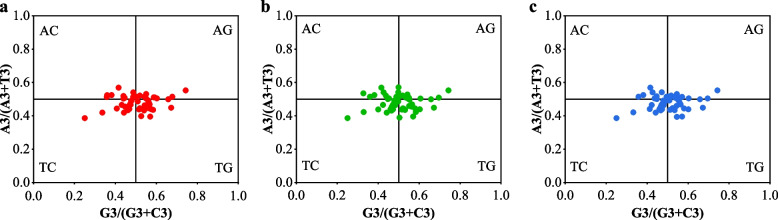
PR2-plot of chloroplast genomes of three Coffea species. **a** *Coffea arabica* **b** *Coffea canephora* **c** *Coffea liberica*

The PR2-plot was employed to investigate the impact of mutation and natural selection on codon usage bias by analyzing the utilization of A/T and G/C at the third position of codons. As shown in Fig. 2, a majority of genes in the three types of coffee were situated in the bottom right region, indicating a higher frequency of T at the third codon position compared to A, and a greater frequency of G compared to C. This suggests that codon usage in the third position of the three coffee types is similar, and that factors influencing codon preference include not only mutation but also natural selection and other unknown elements.

### Neutrality plot

**Fig. 3:**
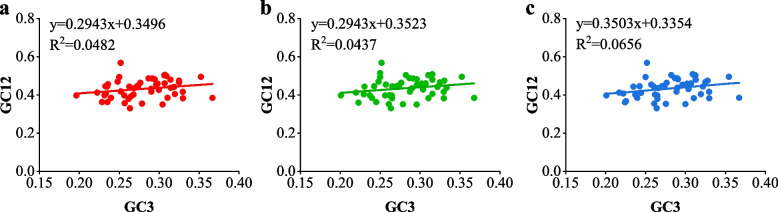
Neutrality plot of chloroplast genomes of three Coffea species. **a** *Coffea arabica* **b** *Coffea canephora* **c** *Coffea liberica*

Neutral analyses based on GC12 and GC3 (Fig. 3) was conducted to quantitatively assess the influence of mutation pressure and natural selection. The slopes of the three regression lines for the three coffee genomes were 0.2943, 0.2943, and 0.3503, respectively, and the correlation coefficients (*r*_1_ = 0.219, *r*_2_ = 0.209, *r*_3_ = 0.256) indicated a weak correlation between GC12 and GC3. These results suggest that mutation pressure accounted for only 29.43%-35.03% of the factors influencing codon usage bias in the three coffee genomes, while natural selection and other factors accounted for 64.97%-70.57%. Thus, mutation pressure had limited effects on codon bias, whereas natural selection played a significant, if not dominant, role. These findings align with the results from the ENc-GC3s and PR2-plot analyses.

### *Correspondence analysis *(*COA*)

**Fig. 4:**
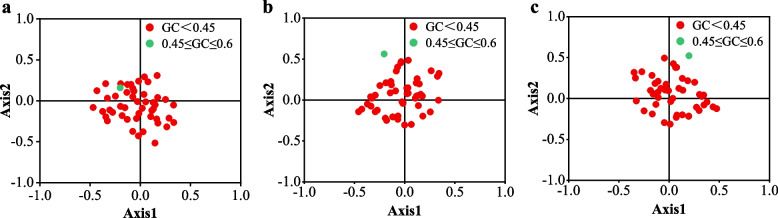
Correspondence analysis of chloroplast genomes of three Coffea species. **a** *Coffea arabica* **b** *Coffea canephora* **c** *Coffea liberica*

Correspondence analysis was conducted to investigate the main factors contributing to variation in coffee codon usage, focusing on the RSCU values. Axis 1 accounted for 18.74%, 18.36%, and 18.98% of the total variation in the three coffee genomes, while axis 2 accounted for 10.15%, 10.79%, and 10.68% of the variation, respectively. The other axes accounted for less than 6.80% of the total variation, indicating that axes 1 and 2 had the most significant influence, particularly axis 1. Additionally, genes were color-coded in red (GC < 0.45) and green (0.45 ≤ GC ≤ 0.60) to investigate the impact of GC content on codon usage bias (Fig. 4). The proportion of genes with different GC contents varied significantly among the three coffee genomes. The majority of genes (98.00%, 98.00%, and 97.91% for *C. arabica*, *C. canephora*, and *C. liberica*, respectively) had GC contents below 45%. Only a small proportion (2.00%, 2.00%, and 4.17%) fell within the range of 45% to 60%. Genes with GC content below 45% were distributed near the center of the axis, while those with GC content between 45 and 60% showed distinct differences, with *C. canephora* located in the upper left and *C. liberica* in the upper right (Fig. 4).

**Fig. 5:**
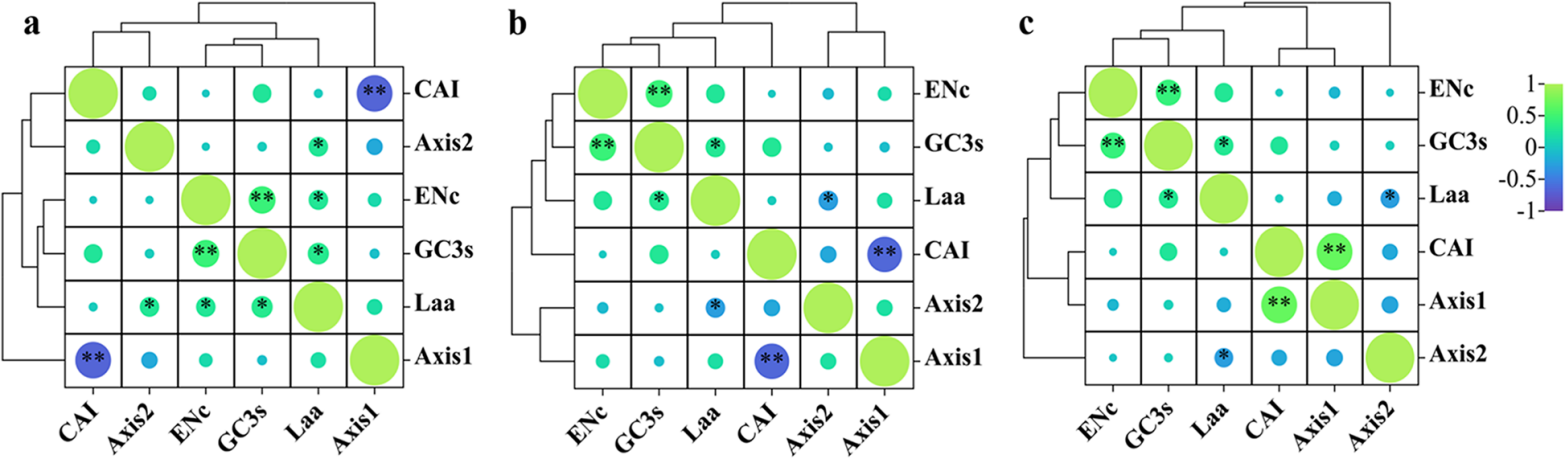
Correlation analysis of axis 1, axis 2 and codon utilization index of chloroplast genomes of three Coffea species. **a** *Coffea arabica* **b** *Coffea canephora* **c** *Coffea liberica*. GC3s indicates the GC content at the third codon position of synonymous codons; ENc represents the effective number of codons; CAI means the codon adaptation index; Laa is defined as the total number of amino acids. * represents* P* < 0.05, ** represents* P* < 0.01

Furthermore, correlation analysis was performed between the codon index and axes 1 and 2 to explore the factors influencing codon usage bias (Fig. 5). In *C. arabica*, the CAI showed a negative correlation with axis 1, while the Laa exhibited a positive correlation with axis 2. In *C. canephora*, CAI had a negative correlation with axis 1, and Laa had a negative correlation with axis 2. In *C. liberica*, CAI showed a positive correlation with axis 1, while Laa had a negative correlation with axis 2. These results suggest that gene expression level and gene length have an impact on codon usage bias in the three coffee species, with gene expression having a greater influence.

## Discussion

Codon is an important link among proteins, amino acids, and genetic material, and its utilization characteristic was of great significance for the protein translation and corresponding functional studies. In the long-term evolution process, organisms gradually evolve into a set of specific codon utilization regulation. A great deal of importance is attached to analyze the preference pattern of genome codons, explore the codons with high frequency and optimal codons, and determine the cause of variation for the research of genetic engineering and genetic evolutionary relationships in plants [30]. In this study, we aimed to gain a detailed understanding of genetic multiformity and codon utilization patterns in the chloroplast genomes of coffee species. In our study, analysis of the codon preference characteristics revealed high similarity among the three coffee species. Base composition analysis showed that all three coffee species had high A/T content and low G/C content, with a preference for A/T-ending codons. Similar patterns have been observed in other plant species, such as *Theaceae*, *Gramineae*, and *Euphorbiaceae* [11–13]. In terms of GC content analysis, the degree of codon preference between large-grained coffee and medium-grained coffee was found to be more similar, indicating a closer relationship between them. Comparative analysis of RSCU values revealed the presence of 30 common preference codons across the three types of coffee, with 96.67% of these codons ending in A/T.

Numerous biological factors can influence codon preference, including gene expression level, gene length, tRNA abundance, mutation preference, and GC content [31–35]. Nevertheless, orthomutation pressure and natural selection have been widely recognized as the dominant factors shaping codon preference in various organisms, explaining inter and intragenic codon usage variations [36, 37], In chloroplast and mitochondrial genomes, natural selection appears to play a particularly significant role in codon preference [38]. In our study, we systematically analyzed the factors affecting coffee codon usage bias in coffee by combining neutrality plots, ENc-plot, and RP2-plot analyses. The PR2-plot analysis represented that codon preference was influenced not merely by mutation, but also by natural selection and other factors. Similar results have been reported in studies of *Arabidopsis thaliana*, *Zea mays*, *Gossypium hirsutum*, and *Glycine max* [39–42]. The ENc-GC3s analysis qualitatively analyzed the major factors of influencing codon usage patterns in the three coffee species. The results revealed that the influence of base mutation pressure on codon usage preference was weaker than that of natural selection and other factors. Similar results have been reported in studies of *Ginkgo biloba*, *Medicago truncatula*, and *Populus tomentosa* [43–45]. Neutrality analysis demonstrated that mutation pressure accounted for only 29.43%-35.03% of the factors influencing codon usage bias, while natural selection and other factors accounted for 64.97%-70.57% in the three coffee species. Natural selection and other factors played a dominant role in codon preference. This was consistent with findings in *Ginkgo biloba*, *Elaeis guineensis*, and *Morus notabilis* [43, 46, 47]. The analysis of codon variation sources revealed that multiple factors influenced codon usage preference in the three coffee species, with natural selection and other factors being the determining factors.

At present, there have been many successful reports on the determination of optimal codons, and codon optimization design to boost the exogenous genes expression in microorganisms, plants, and animals. Determining optimal codon can provide reliable information for reasonable and effective codon modification [48, 49]. In our study, we identified 20, 17, and 19 optimal codons in the three coffee genomes, respectively. These results not only contribute to codon optimization, but also provide insights into the relationship between codon preference and gene expression. When conducting transgenic research, heterologous expression of genes is often needed, and codon usage varies significantly between different species. Therefore, it is essential to explore the codon preferences of exogenous genes and the host system, and modify the codons, adopting the codon conforming to the host genome to improve the transcription and translation efficiency, and gene expression levels [50]. Model plants such as *N. tabacum* and *A. thaliana* are commonly used to study gene function [30]. *E. coli* is often used as a receptor in prokaryotic expression systems, while *S*. cerevisiae is frequently used in eukaryotic systems [41]. Selecting receptors with small differences in codon preference is important for successful expression of exogenous genes and achieving high expression levels [43, 46, 47]. In our study, by comparing the codon usage frequencies of the three coffee genes with those of *A. thaliana*, *N. tabacum*, *E. coli*, and, *S. cerevisiae*, the codon frequency of the three kinds of coffee had slightly difference from that of *N. tabacum* and *S. cerevisiae*. can be used as the hosts for heterologous expression receptors of three coffee genes. To achieve efficient expression of coffee genes in *N. tabacum* and *S. cerevisiae*, it may be necessary to modify some codons with significant preference differences.

## Conclusions

Coffee undeniably plays a crucial role in the tropical agricultural economy global international trade, and human daily life. In this study, bioinformatics was employed to systematically analyze the codon utilization preference characteristics of three coffee genomes. The analysis revealed substantial similarity in codon utilization patterns among these genomes, with a preference for codons ending in A/T. It was determined that codon preference was influenced by natural selection, mutation pressure and other factors, with natural selection being the primary determinant. For heterologous expression receptors of the three coffee genes, *N. tabacum*, and *S. cerevisiae* were evaluated as potential receptor organisms. The consequences of this research hold significant implications for studying the evolution of coffee and enhancing the expression efficiency of exogenous genes. It provided new avenues for genetic transformation in coffee cultivation. On this basis, our team will continue to carry out research on gene function verification, genetic transformation, target gene codon optimization and directional transformation. Do more work for the development of the coffee industry in Yunnan Province.

## Materials & methods

### Genome and sequence selection

The sequence of the complete chloroplast genome of *C*. *arabica* (MN894550.1), *C. canephora* (NC_030053.1), *C. liberica* (MW970411.1) and their coding sequences(CDS) were downloaded and collected from the National Center for Biotechnology Information (NCBI) database (http://www.ncbi.nlm.nih.gov/genbank) on August 15, 2022. The number of raw coding sequences(CDS) of three *Coffea* species chloroplast genomes was 86, 86 and 85, respectively. To avoid error and guarantee the precision of experimental results, the CDS of selected must conform to the following conditions: 1) the sequence length is a multiple of three; 2) each CDS contains a start codon (ATG), a stop codon (TAA, TAG or TGA), and no stop codon in the middle of the sequence. 3) the CDS length must be longer than 300 bp; and 4) the sequence must not be a repeating sequence [51, 52]. Ultimately, the number of carefully selected CDSs of *C. arabica*, *C. canephora*, and *C. liberica* was 50, 50 and 48, respectively. It was utilized to study the subsequent analysis.

### Analysis of codon usage index

The EMBOSS online website (https://www.bioinformatics.nl/cgi-bin/emboss/cusp) was utilized to calculate the GC content of the 1st (GC1), 2nd (GC2), and 3rd (GC3) of the three types of coffee genome sequence codons separately. Additionally, the codon utilization index of three types of coffee genome CDS coding sequences was analyzed by CodonW software (https://codonw.sourceforge.net/), counting the effective number of codons (ENc), codon adaptation index (CAI), GC content at the third codon position of synonymous codons (GC3s), total number of amino acids (Laa) and relative synonymous codon usage (RSCU) [53].

### Analysis of relative synonymous codon usage (RSCU) and relative synonymous codon usage frequency (RFSC)

RSCU is a statistical measure the factual degree of the relative recurrence of each synonymous codon. In case the RSCU value of a particular codon = 1, it implies that each synonymous codon was utilized at the same frequency; RSCU value > 1, it is utilized more frequently than other synonymous codons, and RSCU value < 1, it is utilized less as often as possible than other codons [54].

The RSCU was calculated as follows in Eq. (1):

1 $$\text{RSCU} = \frac{{\text{X}}_{\text{ij}}}{{\sum }_{\text{j}}^{\text{ni}}{{\text{X}}}_{\text{ij}}}{\text{n}}_{\text{i}}$$

A toolkit for scientific researchers integrating various data handling tool (Python) software was used to create the average RSCU values.

Relative synonymous codon usage frequency (RFSC) refers to the ratio of the sum of a codon observed in a test to the whole sum of synonymous codons, which reflects the utilization frequency of each synonymous codon.

The RFSC was calculated as follows in Eq. (2):

2 $$\text{RFSC} = \frac{{\text{X}}_{\text{ij}}}{{\sum }_{\text{j}}^{\text{ni}}{{\text{X}}}_{\text{ij}}}$$

In arrange to choose high-frequency codons, the RFSC comes about of all codons were analyzed. The following principles were executed: RFSC > 60%; or the codon's RFSC is greater than 0.5 times the average of synonymous codons [55, 56].

### Determination of optimal codons

According to the effective number of codons (ENc) value of the genes, 10% of the genes at both ends were selected to establish the gene datasets with high and low expression. The RSCU values of the codons in the two expression libraries were calculated and compared by ΔRSCU (ΔRSCU = high expression of RSCU − low expression of RSCU), and the codons satisfying both conditions of RSCU > 1 and ΔRSCU > 0.08 were determined to be the optimal codons [57].

### Comparative analysis of codon utilization frequency

The extent of codon utilization frequency was used to degree the difference in codon utilization bias. The codon utilization frequency data of *A.* thaliana (http://www.kazusa.or.jp/codon/cgi-bin/showcodon.cgi?species=3702), *N.* tabacum (http:// www.kazusa.or.jp/codon/cgi-bin/showcodon.cgi?species=4097), *E.* coli (http://www.kazusa.or.jp/codon/cgi-bin/showcodon.cgi?sp Ecies = 199,310), and *S.* cerevisiae (http://www.kazusa.or.jp/codon/cgi-bin/showcodon.cgi?species=4932) were downloaded from the Codon Utilization Database (http://www.kazusa.or.jp/codon). These data were compared to the codon utilization frequencies of three types of coffee. Furthermore, three coffee plant species were compared to four model organisms in terms of codon utilization frequency. In case the proportion is less than or equal to 0.5 or greater than equal to 2, it indicates a more prominent distinction in codon utilization preference between two living beings, if the proportion is between 0.5 and 2, which demonstrates that the codon utilization preference is exceedingly comparative, then it can be used as a receptor of heterogenic expression [56].

### Analysis of ENc-plot

The effective number of codons (ENc) is utilized to survey codon use preference at the genome level, and the value of ENc ranges from 20 to 61. GC3s is the GC content at the third codon position of synonymous codons, which is a vital indicator to uncover the inclination of nucleotide specific gravity. The relationship between codon preference and base composition can be uncovered by ENc -plot [52, 58]. To decide whether other factors contribute to codon utilization bias, the value of ENc against GC3s was compiled primarily to uncover the impact of base composition. If abrupt pressure plays a vital part in shaping codon utilization patterns, the ENc values that drop fall within or around the expected curve. The ENc values were much lower than the expected curves when natural selection and other variables impacted codon utilization [52, 58].

### PR2-plot analysis

To analyze the utilization and relationship of purine and pyrimidine at the third codon of the genome sequence, the PR2-plot was plotted with G3/(G3 + C3) as the abscissa and A3/(A3 + T3) as the ordinate. It was assessed whether base mutations impact nucleotide base variation based on the proportions of A, T, G and C base. As long as G and C (or A and T) have comparable proportions, mutation pressure influences codon usage bias completely. When their proportion is too diverse, the natural selection and other variables are likely to affect affecting codon utilization preferences [59, 60].

### Analysis of the neutrality plot

Based on the neutrality plot (GC12-GC3), we were able to estimate and characterize the codon utilization patterns. The neutrality plot was capable of analyzing the correlation between three codon positions quantitatively, thus analyzing the influence variables such as mutation pressure and natural selection [61]. GC12 represents the average GC content of GC1 and GC2, and the plot was made with GC12 as the vertical coordinate and GC3 as the horizontal coordinate. When the regression with a slope of 0 indicates that codon preference is completely affected by natural selection, the regression with a slope of 1, the significant correlation proposes that abrupt pressure may be the only driving force [62, 63].

### Correspondence analysis (COA)

For the investigation of codon utilization patterns, the COA may be a multivariate statistical method for discussing RSCU changes and gene distribution in multidimensional space [64]. In this study, to reflect the variety of codon utilization, the orthogonal axes were produced using 59 codons (except Met, Trp, and three stop codons). The extent of the first axis (Axis 1) represents the greatest impacting figures of codon utilization frequency alteration, and the remaining 58 axes represent the steadily diminishing influencing factor. It is possible to uncover the most variables impacting codon utilization patterns in CDSs by using COA. R software was used to draw the relationship chart of axis 1, axis 2 and the codon utilization index, counting the GC content of the third position of the synonymous codon (GC3s), the number of effective codons (ENc), the total number of amino acids (Laa) and the codon adaptation index (CAI), and further investigate the components affecting codon utilization preference [65].

### Statistical analysis

The CodonW software (https://codonw.sourceforge.net/) was used to analyze the codon characteristic parameters of the three coffee species, including A3s, C3s, U3s, GC, ENc, GC3s, RSCU and CAI. The CUSP in-line program in EMBOSS (https://www.bioinformatics.nl/cgi-bin/emboss/cusp) was used to calculate the codon utilization frequency. The figures in this manuscript was completed by the software of R programming language and origin. Microsoft office Word was used to edit the entire manuscript.

### Supplementary Information

**Additional file 1: Table S1.** RSCU and RFSC values of the codons in chloroplast genomes of three Coffea species.

**Additional file 2: Table S2.** The RSCU values and Δ RSCU value in the high and low expression library of three Coffea species.

**Additional file 3: Table S3. **Comparison of codon usage frequency between three Coffea species and four organisms.

## Data Availability

The chloroplast genome datasets generated and analyzed in this study are available in the NCBI, https://www.ncbi.nlm.nih.gov/nuccore/MN894550.1, https://www.ncbi.nlm.nih.gov/nuccore/NC_030053.1, https://www.ncbi.nlm.nih.gov/nuccore/MW970411.1, and supplementary files.

## Abbreviations

A: Adenine
T: Thymine
C: Cytosine
G: Guanine
GC1,GC2,GC3: The G + C content at the first, second, third codon positions
A3,T3,G3,C3: The content of A,T, G, and C at the third codon position
GC12: The average GC content at the first and second codon positions
RSCU: Relative synonymous codon usage
RFSC: Relative synonymous codon usage frequency
ENc: Effective number of codons
PR2: Parity Rule2
COA: Correspondence analysis
GC3s: GC content at the third codon position of synonymous codons
Laa: Total number of amino acids
CAI: Codon adaptation index
NCBI: National Center for Biotechnology Information
